# *SnoN *expression is differently regulated in microsatellite unstable compared with microsatellite stable colorectal cancers

**DOI:** 10.1186/1471-2407-6-252

**Published:** 2006-10-24

**Authors:** June A Chia, Lisa A Simms, Sarah-Jane Cozzi, Joanne Young, Jeremy R Jass, Michael D Walsh, Kevin J Spring, Barbara A Leggett, Vicki LJ Whitehall

**Affiliations:** 1 The Conjoint Gastroenterology Laboratory, Royal Brisbane and Women's Hospital Foundation Clinical Research Centre and the Queensland Institute of Medical Research, Brisbane, 4029, Australia; 2 The Molecular Cancer Epidemiology Laboratory, The Queensland Institute of Medical Research, Brisbane, 4029, Australia; 3 The Department of Pathology, McGill University, Montreal, H3A 2B4, Canada

## Abstract

**Background:**

*SnoN *is an important regulator of the transforming growth factor beta (TGFβ) signalling pathway and has been shown to exhibit both tumour promotion and suppression activity.

**Methods:**

To further explore the role of this complex molecule in colorectal tumorigenesis, we examined 52 paired normal and tumour colorectal specimens stratified by level of microsatellite instability; 18 with high-level microsatellite instability (MSI-H) and 34 microsatellite stable (MSS). *SnoN *transcript expression was quantitated by real-time PCR and analysed with respect to clinical indicators of prognosis.

**Results:**

Within the MSI-H subgroup, *SnoN *was commonly either up-regulated (6/18, 33%) or down-regulated (7/18, 39%). A significantly different distribution of *SnoN *expression was observed in MSS cancers compared with MSI-H (P ≤ 0.001). Whilst 17/34 (50%) of MSS tumours demonstrated up-regulation, none showed down-regulated expression. Within the MSI-H subgroup, up-regulation was significantly correlated with lack of repeat tract mutation in the *TGFβRII *gene (P ≤ 0.025), suggesting that *SnoN *is more frequently up-regulated in the presence of functional TGFβ signalling.

**Conclusion:**

Together these data support the notion that SnoN has both oncogenic and tumour suppressive properties depending on other genetic changes within the tumour, and that the MSI-H pathway of colorectal tumorigenesis presents an excellent model for the study of these opposing functions.

## Background

*SnoN *(*ski*-*related novel gene*) is a member of the highly homologous *Ski *gene family [1], and can act as a repressor of the TGF-β signalling pathway through either SnoN homodimerisation or heterodimerisation with Ski [1-6]. This repression is mediated through binding of SnoN to SMAD proteins, which are responsible for the propagation of signals initiated by TGF-β ligand for the transcription of genes required for cell growth control and differentiation [1,4,7,8]. Conversely, SnoN has also been shown to suppress tumour growth both in a heterozygote mouse model of *SnoN *inactivation [9] and in human cells [10]. These opposing functions have led to difficulty in interpreting the complex roles of SnoN in human malignancies.

Both *SnoN *and *Ski *have been implicated in the development of human cancers including colon [11], breast [12], melanoma [3] and oesophageal cancers [13]. Expression of *SnoN *has not previously been examined in colorectal cancer (CRC), although a study of gene copy number status showed that the *SnoN *locus was partially or completely deleted in 55% of CRCs and amplified in 15% [11]. Interestingly, increased copy number of *Ski *was found to be a negative prognostic indicator whilst no correlation with patient outcome was observed for *SnoN*. Since regulation of TGFβ signalling by SnoN involves a combination of proteolytic degradation and transcriptional up-regulation in addition to changes in copy number, we suggest that quantification of *SnoN *expression may be a more informative way to assess its role in colorectal tumour development.

Colorectal tumours stratified by level of microsatellite instability represent two principal pathways of sporadic colorectal tumorigenesis. The majority of sporadic CRCs are microsatellite stable (MSS) and aneuploid. These have been well studied but are understood to be heterogeneous at the molecular level [14]. Cancers displaying a high level of microsatellite instability (MSI-H) comprise approximately 15% of all sporadic CRCs, are diploid and generally have a favourable prognosis [15]. Whilst the major mechanisms for gene inactivation in MSS cancers are mutation and deletion, MSI-H tumours progress predominantly due to repeat-tract mutation and aberrant promoter hypermethylation of important target tumour suppressor genes [16].

We have examined the frequency of altered *SnoN *expression in a series of CRCs stratified by MSI-status. Based on our findings we propose that MSI-H cancers present an excellent model for studying the dual roles of *SnoN *in both tumour suppression and promotion.

## Methods

### Patient specimens

Fifty-two paired normal and tumour tissue specimens were obtained in a fresh state from colectomy specimens surgically resected at the Royal Brisbane and Women's Hospital between 1993 and 2005. The Human Research Ethics Committee of the Queensland Institute of Medical Research and the Royal Brisbane and Women's Hospital Research Ethics Committee approved this study and all patients provided written informed consent. The standard panel of markers (*BAT25*, *BAT26*, *D5S346*, *D2S123 *and *D17S250*) recommended by a National Cancer Institute workshop were used to determine the MSI-status of the cancers, as previously described [17]. Patients were classified as microsatellite instability high (MSI-H) if >30% of markers showed instability. The remainder of patients were microsatellite stable (MSS). The patient cohort was enriched for MSI-H tumours, but was otherwise unselected. Patients with familial adenomatous polyposis (FAP) or hereditary non-polyposis colorectal cancer (HNPCC) were excluded from the study.

### Expression analysis

Total RNA was extracted from tissue samples which had been snap frozen in liquid nitrogen then stored at -80°C using the QIAGEN RNeasy Midi Kit. Total RNA (5 μg) was reverse transcribed using random hexamers and SuperScript III Reverse Transcriptase (Invitrogen, USA). Semi-quantitative real-time polymerase chain reaction was performed using a Corbett Rotor-Gene 3000 machine (Corbett Research, Australia) with the following gene specific primers: Reference gene, β-ActinF: 5'-TCATGAAGTGTGACGTGGACATC-3'; β-ActinR: 5'-CAGGAGGAGCAATGATCTTGATCT-3'; Gene Of Interest, SnoNF: 5'-CACCCCAGCTACTACTTATAC-3', SnoNR: 5'-TTGCCTCTGTCTTTGTGAGC-3') and Platinum SYBR Green qPCR Supermix-UDG (Invitrogen, USA). Each reaction contained 8 μL of qPCR mix, 5 pmol of each forward and reverse primer and 4 μL of diluted cDNA template. The following cycling conditions were applied: 50°C for 2 min, 95°C for 2 min, followed by 45 cycles of 95°C for 15 sec, 59°C for 15 sec, 72°C for 20 sec, 76°C of 15 sec. Data for each cycle was acquired after the final cycling step. A melt ramp consisting of 1°C increments from 60°C to 99°C (30 sec first step, 1 sec for subsequent steps) followed the 45 cycles. Each reaction was carried out in duplicate (patient samples) or triplicate (calibrator). The relative expression of SnoN to β-Actin was calculated using the method previously described by Pfaffl [18]. qPCR results from each set of reaction replicates varied no more than 5% from the mean. Normalised *SnoN *expression values (R) for all tumours were divided by the R value for their matched normal mucosa to indicate fold up-regulation, the inverse gave a whole number to indicate fold down-regulation. Data was analysed based on empirical fold differences (student's T-test) as well as number of tumours showing greater than 2 fold up- or down-regulation (Pearson's chi square). The cut-off of 2-fold was arbitrarily chosen as significant, based on previous reports in the colorectal literature [19,20].

### TGFβRII mutation analysis

The presence of somatic mutations in the poly(A)_10 _tract of the *transforming growth factor β receptor II *(*TGFβRII*) coding sequence was investigated in MSI-H tumours by PCR followed by direct manual sequencing of the adenosine nucleotide. Both 1-bp and 2-bp deletions are predicted to result in a truncated protein product. The PCR consisted of a 50 μL reaction containing 250 ng template DNA, 1.5 mM MgCl_2_, 0.2 mM nucleotides, 20 pmol forward and reverse primers, 1X Gold Buffer and 2U AmpliTaq Gold (Applied Biosystems). Primers were TGFBR2f: 5'-CTTTATTCTGGAAGATGCTGC-3' and TGFBR2r: 5'-GATGATGTTGTCATTGCACTC-3'. Following column purification (QIAGEN) 1 μL of PCR product was subjected to direct manual sequencing using the same forward and reverse primers with the Ampli-Cycle cycle sequencing kit (Perkin Elmer). The 115-bp product was electrophoresed on a 6% denaturing polyacrylamide gel for 75 minutes.

### Clinical correlates

Disease-staging data was obtained for all patients according to the ACPS [21] and TNM staging systems [22]. TNM staging provided information on the extent of primary tumour invasion (T), local lymph node spread (N) and metastasis to a distant site (M). Patients were followed for an average of 43 months and clinical data was recorded for patient age, sex and tumour anatomical location. Tumour recurrence, death due to cancer and death due to any other cause were also noted.

### Statistical analysis

Tabular data were analysed by Pearson's chi square using Web Chi Square Calculator [23] to determine significance. Non-parametric t-tests (Mann-Whitney) were performed using GraphPad Prism version 4.00 for Windows [24]. P-values less than 0.05 were considered statistically significant.

## Results

### SnoN expression levels compared with MSI-status

**Figure 1:**
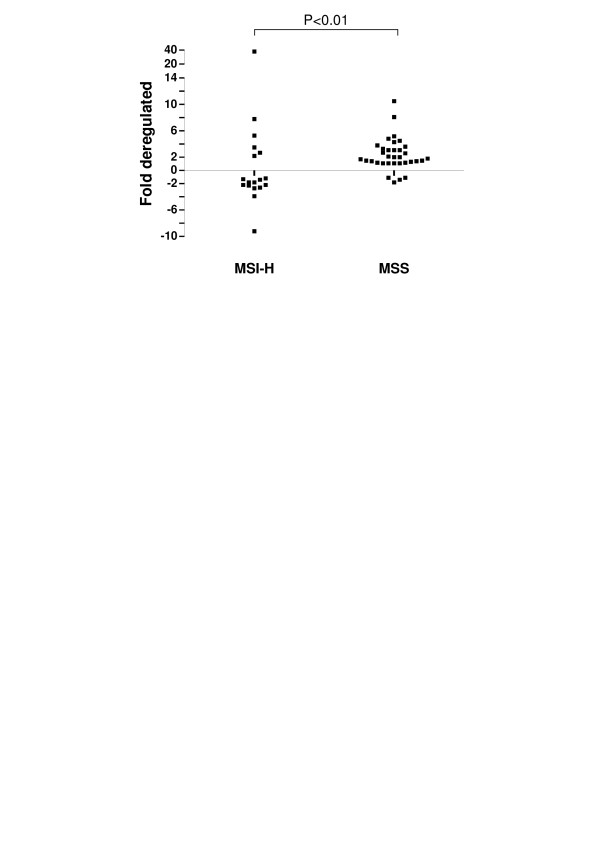
Distribution of *SnoN *expression in tumours stratified by MSI-status. *SnoN *data points were determined by semi-quantitative real-time PCR and are expressed as either fold up- or down-regulated, as compared to matched normal mucosa. Whilst there was a wide range of increased and decreased expression changes in the MSI-H subgroup, no MSS tumours demonstrated down-regulation greater than 2 fold.

Transcript levels of *SnoN *were examined in 34 MSS and 18 MSI-H tumours relative to matched normal mucosa. There was a significantly different distribution of expression observed in MSI-H compared to MSS cancers (P < 0.01). Whilst expression varied from 9.2 fold down-regulated to 38.1 fold up-regulated in MSI-H cancers, the majority of MSS cancers showed expression higher than matched normal mucosa levels, to a maximum of 10.5 fold (Fig. 1). Considering >2 fold expression differences as significant, *SnoN *was up-regulated in 6/18 (33%) MSI-H cancers, whilst expression was down-regulated in 7/18 (39%) tumours in this group. By contrast, within the MSS tumour group, cancers were equally likely to demonstrate *SnoN *up-regulation (17/34, 50%) as they were to show no change in expression level (17/34, 50%) (Table 1, P < 0.001). Significance was maintained even when the cut-off was reduced to 1.5 fold de-regulation (P < 0.001).

### SnoN expression levels compared with clinical features

No significant correlations were observed between *SnoN *expression levels and patient age, sex, site or ACPS stage (Table 1). Evaluation of extent of tumour invasion (T), local spread to lymph nodes (N) or distant metastatic spread (M) derived from the TNM staging system did not reveal any significant correlations. Within the MSS group, more patients without increased *SnoN *expression had a stage D tumour, as compared with those patients with increased *SnoN *expression (Table 1). Of patients with a stage A, B or C tumour, 3 had a recurrence of cancer. None of these tumours showed increased *SnoN *expression. When these two groups were combined to indicate a poor prognosis, there was a statistically significant association between poor outcome and failure to elevate *SnoN *(Table 1, P < 0.025). There were only two stage D MSI-H tumours, neither of which showed increased *SnoN *expression.

### TGFβRII mutation

Fifteen MSI-H tumours were assessed for repeat tract mutation in the coding region of the *TGFβRII *gene (Table 2). Mutations were detected in 12/15 (80%). Interestingly, three out of the six tumours demonstrating up-regulated *SnoN *expression were wild-type for this usually common mutation, whilst all nine tumours which did not have *SnoN *up-regulated had a mutation of either one or both *TGFβRII *alleles (P ≤ 0.025).

## Discussion

SnoN is an important negative regulator of TGFβ induced tumour suppression, and exerts an oncogenic effect by binding to SMAD protein complexes [7,25]. Importantly, SnoN has also been shown to suppress tumour growth in both epithelial cell line and murine models [9,10]. To further delineate the role of *SnoN *gene regulation in colorectal tumours, we quantified mRNA expression in a series of sporadic CRCs stratified by MSI-status.

Down-regulation of a gene in a significant number of tumours compared with matched normal mucosa would usually suggest abrogation of tumour suppressor gene function. Strikingly, *SnoN *was down-regulated in 39% of MSI-H CRCs in this study (7/18), but not in any of the 34 MSS cancers examined (P ≤ 0.001). This suggests that *SnoN *may be an important tumour suppressor gene, exclusively in CRCs progressing via the MSI pathway of tumorigenesis. Even when the arbitrary cut-off for significant expression differences was reduced to only 1.5 fold, the statistically significant association between up-regulation of *SnoN *in MSS CRCs and down-regulation in MSI-H CRCs was maintained (P ≤ 0.001), suggesting that even subtle changes in *SnoN *gene expression levels are capable of exerting a functional effect.

There is currently no evidence that the *SnoN *locus is a direct target for down-regulation. Although repeat-tract mutation and promoter hypermethylation are the major mechanisms of gene disruption in MSI-H CRCs, the *SnoN *transcript does not contain an extended repeat tract, nor does the 5' region represent a typical CpG island likely to be silenced by methylation. Whilst chromosomal instability is a characteristic feature of most MSS cancers, locus deletion is an uncommon mechanism for gene down-regulation in MSI-H tumours. It is more likely that *SnoN *is transcriptionally down-regulated due to disruption of the negative feedback loop involving TGFβ signalling [7].

Accumulating evidence suggests that SnoN is an important regulator of TGFβ signalling, and acts predominantly through binding to TGFβ signal-transducing SMAD proteins [7,25]. High levels of SnoN have been shown to inhibit TGFβ signal transduction in breast [12] and skin cells [26]. In MSI-H tumours, mutation of *TGFβRII *causes resistance to TGFβ-induced growth inhibition [27]. We proposed that such a situation would decrease the selection pressure to up-regulate *SnoN *and therefore examined the A_10 _repeat tract mutation of *TGFβRII *commonly observed in MSI-H cancers [28]. We found that *SnoN *was indeed up-regulated more commonly in tumours with a wild-type receptor sequence (P ≤ 0.025), which may support a link between intact TGFβ signalling and *SnoN *up-regulation.

*SnoN *was also up-regulated compared to matched normal mucosa in a large number of MSS cancers (17/34, 50%), however reduced expression was not observed for any MSS tumour. This is discordant with the observed frequencies of gene amplification (15%) and deletion (55%) reported by Buess and colleagues [11], and highlights the importance of examining expression levels where post-transcriptional processing may further modulate transcript levels beyond the linear affect of altered copy number. Chromosomal instability is common in MSS cancers, and usually manifests as high rates of deletion of chromosomal regions where a mutated tumour suppressor gene is located on the remaining allele. It is possible that the high rate of deletion observed by Buess and colleagues is a result of another gene target on chromosome 3q26, whilst expression of the remaining *SnoN *allele is increased through compensatory transcriptional mechanisms, despite hemizygous deletion.

Luo (2004) suggested that the seemingly opposing tumour promoting and suppressing roles of SnoN may reflect the likewise dual roles of TGFβ in tumorigenesis [1]. It was proposed that early in tumour development TGFβ signalling would be activated to facilitate growth suppression. However, subsequent disruption of TGFβ signalling later in tumorigenesis could enhance the ability of TGFβ to promote tumour invasion and metastasis. Therefore, abrogation of TGFβ signalling via up-regulation of *SnoN *could result in tumours which are large but less likely to metastasise. This is entirely consistent with our finding of a more positive prognostic outcome in relation to cancers with increased *SnoN *expression. MSI is a well-established marker for a favourable prognosis [29]. This is consistent with only 2/18 of the MSI-H tumours in this series being ACPS stage D. Furthermore, it is interesting to note that neither of these cancers showed up-regulation of *SnoN*.

Our suggested association between failure to up-regulate *SnoN *and poor patient outcome in MSS cancers may be related to the role of SnoN in TGFβ signalling. This interesting observation warrants further investigation in a larger patient cohort which would allow correction for tumour stage. Comprehensive studies are now necessary to fully elucidate the connection between disrupted TGFβ signalling and the role SnoN may play in maintaining the balance between cell proliferation and differentiation.

## Conclusion

*SnoN *transcript expression was commonly up-regulated in colorectal tumours, regardless of microsatellite instability status. Importantly, *SnoN *was also frequently down-regulated specifically in microsatellite instability high (MSI-H) colon cancers. Based on this finding, we propose that MSI-H tumours present a unique model for examining the dual suppressive and oncogenic roles of this important regulatory gene in colorectal tumorigenesis.

## Competing interests

The author(s) declare that they have no competing interests.

## Authors' contributions

JC carried out the quantitative real-time PCR assays and analysis and contributed to drafting of the manuscript. LS performed the *TGFβRII *mutation analysis. SC contributed to the real-time PCR assays and analysis. MW assisted with cohort selection and clinical analysis. JY contributed to study design and review of the manuscript. JJ contributed to study design and interpretation of results in the context of TGFβ signalling. KS participated in study design and review of the manuscript. BL contributed to study conception and design and collected all clinical data. VW contributed to study conception, data analysis and drafting of the manuscript. All authors read and approved the final manuscript.

## Pre-publication history

The pre-publication history for this paper can be accessed here:



## Figures and Tables

**Table 1:** *SnoN *expression differences relative to MSI-Status and Clinical Characteristics

	***MSI-H***				***MSS***			
	*SnoN *Expression Level				*SnoN *Expression Level			

	Total	Increased	No Change	Decreased	Total	Increased	No Change	

**Total No. Patients**	18	6	5	7	34	17	17	P < 0.001^Δ^
Sex								
Male	6	2	1	3	21	9	12	
Female	12	4	4	4	13	8	5	
Site								
Proximal	13	3	5	5	14	8	6	
Distal	5	3	0	2	20	9	11	
ACPS Stage								
A	5	0	4	1	6	3	3	
B	8	5	0	3	15	8	7	
C	3	1	0	2	5	3	2	
D	2	0	1	1	7	2	5	
Recurrence of Cancer in ACPS Stage A, B or C	0	0	0	0	3	0	3	P < 0.1•
ACPS Stage D *or *Recurrence of Cancer in ACPS Stage A, B or C	2	0	1	1	10	2	8	P < 0.025◆

^Δ^*SnoN *expression relative to MSI-status

^• ^*SnoN *expression relative to recurrence in ACPS stage A, B or C in MSS subgroup

^◆ ^*SnoN *expression relative to the number of patients with an ACPS stage D MSS cancer, or ACPS stage A, B or C MSS cancer which recurred

**Table 2:** TGFβRII mutation status relative to *SnoN *expression

	**SnoN Expression Level in MSI-H Cancers**			
	Total	Increased	No Change	Decreased

**Total No. Patients**	15	6	4	5
TGFβRII Mutated	12	3	4	5
TGFβRII Wild Type	3	3	0	0
